# The Checkpoint Regulator SLAMF3 Preferentially Prevents Expansion of Auto-Reactive B Cells Generated by Graft-vs.-Host Disease

**DOI:** 10.3389/fimmu.2019.00831

**Published:** 2019-04-17

**Authors:** Ninghai Wang, Burcu Yigit, Cees E. van der Poel, Marta Cuenca, Michael C. Carroll, Roland W. Herzog, Pablo Engel, Cox Terhorst

**Affiliations:** ^1^Division of Immunology, Harvard Medical School, Beth Israel Deaconess Medical Center, Boston, MA, United States; ^2^Program in Cellular and Molecular Medicine, Harvard Medical School, Boston Children's Hospital, Boston, MA, United States; ^3^Herman B Wells Center for Pediatric Research, Indiana University School of Medicine, Indianapolis, IN, United States; ^4^Immunology Unit, Department of Cell Biology, Immunology and Neurosciences, Medical School, University of Barcelona, Barcelona, Spain

**Keywords:** SLAMF3, cGVHD, B cells, autoreactive, alloimmunity

## Abstract

Absence of the mouse cell surface receptor SLAMF3 in SLAMF3-/- mice suggested that this receptor negatively regulates B cell homeostasis by modulating activation thresholds of B cell subsets. Here, we examine whether anti-SLAMF3 affects both B and T cell subsets during immune responses to haptenated ovalbumin [NP-OVA] and in the setting of chronic graft vs. host disease (cGVHD) induced by transferring B6.C-*H2*^*bm*12^/KhEg (bm12) CD4^+^ T cells into B6 WT mice. We find that administering αSLAMF3 to NP-OVA immunized B6 mice primarily impairs antibody responses and Germinal center B cell [GC B] numbers, whilst CXCR5^+^, PD-1^+^, and ICOS^+^ T follicular helper (TFH) cells are not significantly affected. By contrast, administering αSLAMF3 markedly enhanced autoantibody production upon induction of cGVHD by the transfer of bm12 CD4^+^ T cells into B6 recipients. Surprisingly, αSLAMF3 accelerated both the differentiation of GC B and donor-derived TFH cells initiated by cGVHD. The latter appeared to be induced by decreased numbers of donor-derived Treg and T follicular regulatory (TFR) cells. Collectively, these data show that control of anti-SLAMF3-induced signaling is requisite to prevent autoantibody responses during cGVHD, but reduces responses to foreign antigens.

## Introduction

The signaling lymphocyte activation molecule family (SLAMF) of cell surface receptors, which consists of nine trans-membrane proteins (SLAMF1-9) serve as co-stimulatory molecules at immune synapses, are involved in viral and bacterial recognition and modulate myeloid and lymphocyte development (1). We previously found that the homophilic receptor SLAMF6 (Ly108, NTB-A) is implicated in the Germinal Center Reaction and that monoclonal antibodies directed against SLAMF6 reduce antibody responses to foreign antigens (Ags) and affect the number of auto-reactive B cells (2, 3). SLAMF3 (CD229. Ly9) is also localized at the interface between the immune synapse, suggesting a role for SLAMF3 in regulating humoral immune responses (1, 4).

Our studies with SLAMF3 deficient mice demonstrated that transitional 1, MZ, and B1a B cells were markedly expanded, whereas the development of conventional B-lymphocytes was unaltered (5). As MZ and B1 B cells respond to foreign Ags more rapidly than conventional B cells, elevated levels of IgG3 natural Abs were found in the serum of SLAMF3-deficient mice. Furthermore, a striking increase of T-independent Abs after immunization with 2,4,6-trinitrophenyl-Ficoll was found (5). Administering a mouse monoclonal antibody (mAb) directed against murine SLAMF3 (αSLAMF3) selectively eliminated splenic MZ B cells and significantly reduced the numbers of B1 and transitional 1 B cells in wild-type mice. Surprisingly, administering an agonistic anti-SLAMF3 mAb (Ly9.7.144) diminished both T cell-dependent and –independent antibody responses indicating a role for SLAMF3 dependent signaling in negative regulation of humoral immune responses. Anti-SLAMF3 (Fab')2 has a similar effect, which excludes Fc dependent effector mechanisms. The concept of SLAMF3 as negative regulator of antibody responses was further supported by the finding that aged SLAMF3-deficient mice developed spontaneous autoantibodies against nuclear antigens (5, 6).

Here, we examine whether αSLAMF3 affects both B and T cell subsets during immune responses to haptenated ovalbumin [NP-OVA] and in the setting of chronic graft vs. host disease (cGVHD) induced by transferring B6.C-*H2*^*bm*12^/ KhEg (bm12) CD4^+^ T cells into B6 WT mice (7–9). We find that administering a single dose of αSLAMF3 to NP-OVA immunized B6 mice primarily impairs antibody responses and Germinal center B (GC B) cell numbers, whilst CXCR5^+^PD-1^+^, and ICOS^+^ T follicular helper (TFH) cells are not significantly affected. By contrast, two injections of αSLAMF3 markedly enhanced autoantibody production upon induction of cGVHD by the transfer of bm12 CD4^+^ T cells into B6 recipients (8, 9). Surprisingly, αSLAMF3 accelerated both the differentiation of GCB and donor-derived TFHs cell initiated by cGVHD. The latter appeared to be induced by expansion of donor-derived regulatory T (Treg) and T follicular regulatory (TFR) cells. Our findings suggest that, SLAMF3 induced signaling plays a different role in the setting of normal immune responses and in response to autoantibody responses.

## Materials and Methods

### Mice

C57BL/6 (B6) WT and B6.C-2^bm12^/KhEg (*bm12*) mice were obtained from the Jackson Laboratory. B6.C-2^bm12^/KhEg (*bm12*) x CD45.1 mice were generated by crossing B6.C-2^bm12^/KhEg (*bm12*) with CD45.1.B6. Experiments were conducted using age-matched 8–10 weeks old female mice. All animals are maintained under specific pathogen-free conditions at the Beth Israel Deaconess Medical Center (BIDMC) animal facility. Experiments were performed according to the guidelines of the Institutional Animal Care and Use Committee (IACUC) at BIDMC.

### Mouse Anti-SLAMF3 Antibody

Mouse anti-mouse SLAMF3 (SLAMF3.7.144) (IgG1 isotype) monoclonal antibody was generated as described elsewhere (10).

### NP-OVA Immunizations

NP-OVA immunizations and measurement of NP-specific antibodies are described elsewhere (2). In short, WT mice were immunized with 50 μg NP-OVA in Complete Freund's Adjuvant (CFA). At the same time, mice were injected with 250 μg/mouse αSLAMF3 or mIgG1 isotype control. Immunized mice were sacrificed on day 9. Cell subsets analyzed from splenocytes: TFH cells: CD4^+^CXCR5^+^PD1^+^; GC B cells: B220^+^GL-7^+^FAS^+^.

### cGVHD Induction by the Transfer of *bm12* CD4+ T Cells Into B6 Mice

We adapted the *bm12* transfer model, as originally described by Morris et al. (9). Eight to ten weeks old B6 WT mice were injected *i.p*. with 6 × 10^6^ purified CD4^+^ T cells from *bm12* or *bm12*xCD45.1 mice.

For *in vivo* anti-SLAMF3 injections, recipients were injected *i.p*. with 200 μg of anti-SLAMF3 antibody or IgG1 isotype control on days−1 and 14 after transfer of 3 × 10^6^ *bm12* CD4^+^ T cells into B6.WT mice. Mice were sacrificed and analyzed on day 28.

### Flow Cytometry

Single-cell suspensions were prepared from spleens using standard procedures. After red blood cell (RBC) lysis (Sigma, St. Louis, MO), single cell suspensions were obtained. Cells were blocked with anti-CD16/32 Ab (2.4G2, Biolegend) and stained in FACS staining buffer (2.5% FBS, 0.05% sodium azide in PBS). The following antibodies were used: CD4 (L3T4), CD44 (IM7), CD62L (MEL-14, CD69 (H1.2F3), CD86 (GL-1), CD138 (281-1), B220 (RA3-6B2), CD19 (6D5), FAS (Jo2), T-and B-cell activation antigen (GL-7), CXCR5 (2G8), and PD-1 (29F, 1A12) were purchased from eBioscience (ThermoFisher, Cambridge, MA), BD Biosciences (Woburn, MA), or Biolegend (San Diego, CA). TFH cells were stained as previously described (2). Dead cells were excluded with 4,6-Diamidino-2-phenylindole (DAPI). Data were acquired on a BD LSR II cytometer and analyzed using FlowJo software (Tree Star, Ashland, Oregon).

### Intracellular Cytokine Staining

Cytokine production was assessed with BD Cytofix/Cytoperm containing BD Golgi-Plug (BD Biosciences). Cells were stimulated with phorbol 12-myristate 13-acetate (PMA, 50 ng/ml, Sigma), Ionomycin (1 μg/ml, Sigma), and GolgiStop (1 μl/ml, BD Biosciences) at 37°C in 5% CO_2_ for 4 h. After surface staining, cells were fixed, permeabilized, and stained for IFN-γ (PE-anti-mouse IFN-γ, Biolegend), IL-4 (PE-anti-mouse IL-4, Biolegend), and IL-17 (PE-anti-mouse IL-17A, Biolegend). For intracellular staining IL-21, permeabilized cells were incubated with IL-21R/Fc chimera (R&D systems) for 1 h at 4°C. Cells were then washed and stained with PE-conjugated affinity-purified F(ab')_2_ fragment of goat anti-human Fc γ antibody (Jackson ImmunoResearch Laboratories) for 30 min at 4°C. Viability was assessed using LIVE/DEAD Cell Viability Assays (Life Technologies).

### ELISA

Titers of anti-nucleosome antibodies in the serum were determined by ELISA as described previously (11, 12). In brief, met-BSA-precoated Immunolon plated were coated overnight with double stranded DNA (dsDNA) and then with total histone solution. Samples were incubated on plates in various dilutions between 1:600 and 1:1,200, and then washed, and autoantibodies were detected with anti-mouse IgG-HRPO (GE Healthcare).

Autoantibody titer was expressed as ELISA unit, comparing OD values of samples with a standard curve prepared with serial dilutions of ANA-positive NZM2410 serum pool. Anti-chromatin and anti-dsDNA titers were determined as for the anti-nucleosome levels. UV-irradiated Immunolon plates were incubated overnight with 3 μg/ml chicken chromatin (13) or mung bean nuclease (New England Biolabs, Ins.)-treated dsDNA (Sigma-Aldrich. Anti-single-stranded DNA (ssDNA) was determined as describe previously (14).

### Statistical Analysis

Statistical significance was determined by unpaired *t*-test (two-tailed with equal SD) using Prism software (GraphPad, San Diego, CA, USA). The *p* < 0.05 was considered statistically significant.

## Results

### Administering αSLAMF3 Reduces GC B Cell Formation and Antibody Resposes to NP-ovalbumn

**Figure 1:**
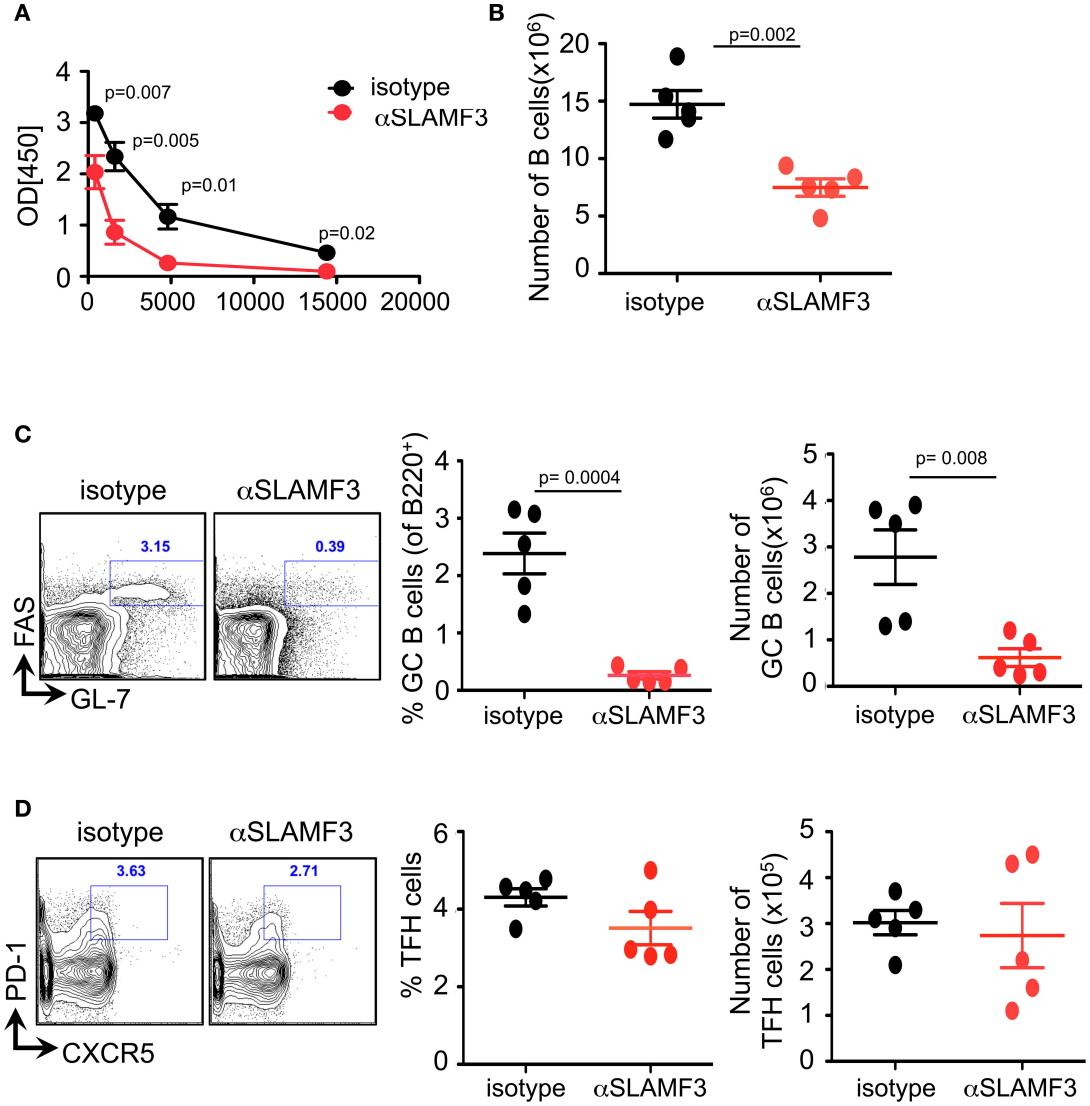
Administering αSLAMF3 to NP-OVA immunized B6 WT mice reduces B cell numbers and antibody responses. WT mice were immunized with NP-OVA in CFA along with 200 μg/mouse αSLAMF3 or isotype IgG1. Nine days later mice were euthanized and spleens were analyzed. **(A)** NP-specific antibody titers from serum of αSLAMF3 and isotype injected mice are as shown. **(B)** Total number of splenocytes from αSLAMF3 and isotype injected mice. **(C)** Representative Flow cytometry plots for GC staining: CD19^+^GL-7^+^FAS^+^ B cells (left), percentage and numbers of GC B cells (right). **(D)** Representative Flow cytometry plot showing gating strategy for TFH cells: CD4^+^PD-1^+^CXCR5^+^ (left panel) Percentages and numbers of TFH cells in spleen of αSLAMF3 and isotype injected mice (right panel). Data representative of three independent experiments. *P*-values are as shown.

To assess which cell types are affected by αSLAMF3 we immunized B6. WT mice with NP-OVA in conjunction with injecting αSLAMF3 or an isotype control. On day 9 we found no difference in spleen weight or total number of splenocytes between isotype and αSLAMF3 injected groups (Figure S1). As expected from a preliminary study (6), we found significantly reduced levels of NP-specific antibodies in the serum of αSLAMF3 injected groups as compared to isotype-injected mice (Figure 1A). Further analysis revealed a significant reduction in total B cells and MZ B cells (Figure 1B and Figure S1), but more importantly dramatically reduced percentage and numbers of GC B cells in spleen of αSLAMF3 injected mice (Figure 1C). However, no difference in total CD4^+^ T cells or TFH cells was found (Figure 1D and Figure S1), suggesting that the antibody primarily affects B cells in this system. While this was in the case of co-injection of αSLAMF3 together with NP-OVA immunization, injection of antibody at a later time point (day 4) showed similar results (Figure S2), demonstrating that our findings are independent of time of injection.

### Administering αSLAMF3 Enhances Autoantibody Production Upon Induction of cGVHD by the Transfer of bm12 CD4^+^ T Cells Into B6 Recipients

**Figure 2:**
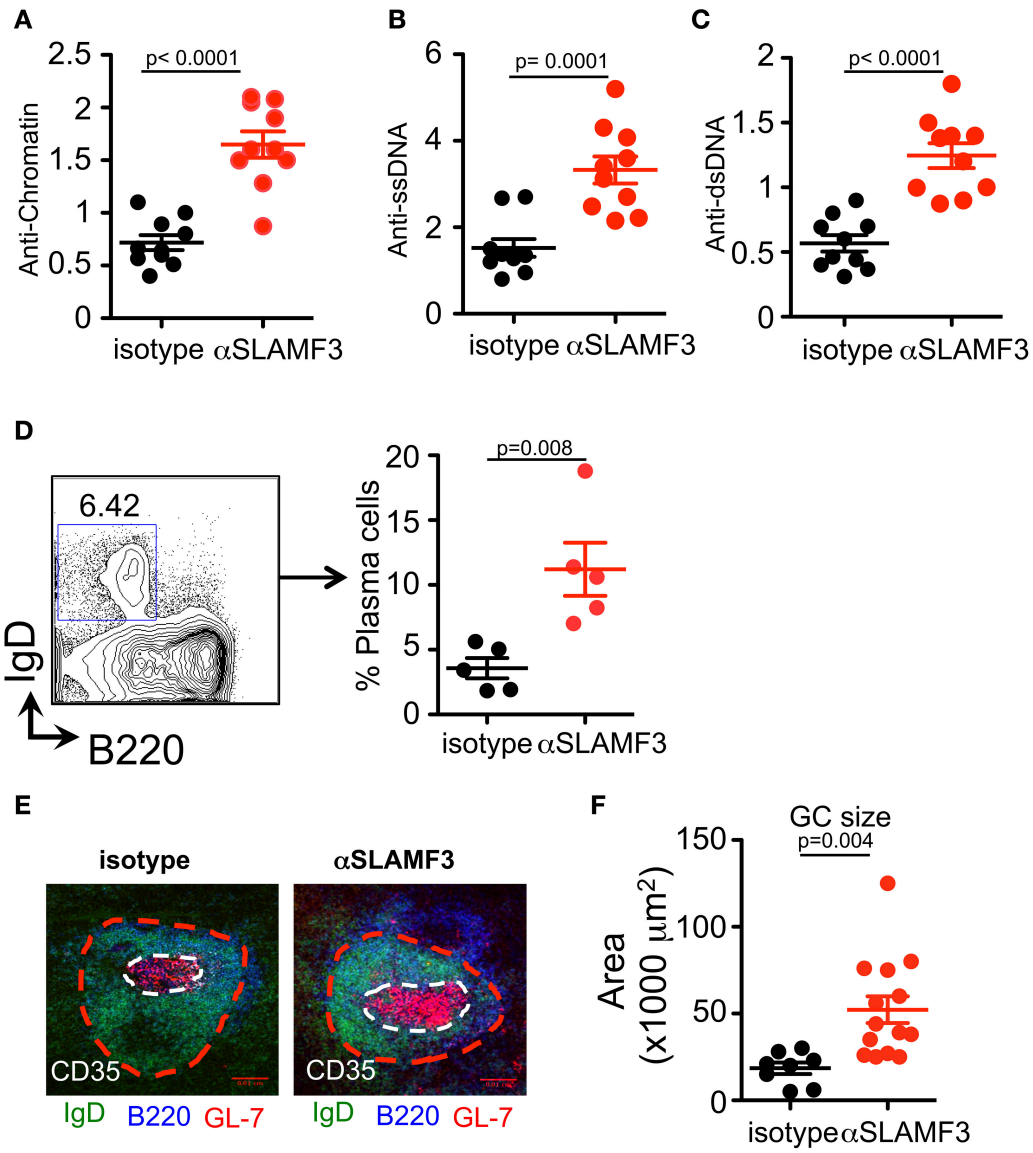
Administration of αSLAMF3 induces lupus-related autoantibody responses in the recipients of bm12 CD4^+^ T cells. 6 × 10^6^ CD4^+^ T cells isolated from *bm12* female mice were transferred into B6 WT recipients by *i.p*. injection. The recipients of bm12 CD4^+^ T cells were injected with anti-SLAMF3 or Isotype IgG1 (200 μg/mouse) on days−1 and 14. Mice were euthanized on day 28. Spleens and serums were analyzed. **(A–C)** Anti-Chromatin, anti-ssDNA, and anti-dsDNA in the serum of recipient mice were determined by ELISA. **(D)** Representative FACS plots showing B220^+^IgD^−^CD138^+^ plasma cells from the spleens (Left panel). Percentage of plasma cells in spleen of isotype and αSLAMF3 injected mice (Right panel). **(E,F)** The indicated recipient mice were sacrificed and spleen were embedded in optimal cutting temperature compound (OCT) tissue media and frozen on dry ice. Seven-micrometer thick frozen sections were fixed to slides in ice-cold acetone for 15 min. The sections were stained with B220 FITC, GL-7 PE, and CD35 Pacific Blue. Representative confocal images and quantification of the germinal center sizes are shown. Data represent at least four independent experiments, *p*-values are as shown.

Our previous studies demonstrated that spontaneous anti-nuclear antibody (ANA) production in SLAMF3-/- mice was independent of the background (B6.129 or Balb/c.129), suggesting that SLAMF3 may play an important role in regulating autoimmunity (6). These findings led us to investigate the consequences of modulating SLAMF3 during autoimmune responses using a monoclonal mouse anti-SLAMF3 antibody (αSLAMF3). In addition to initiating cGVHD, 200 μg/mouse αSLAMF3 or isotype control was *i.p*. injected into *bm12* mice−1 and 14 days after transfer of B6.WT donor CD4^+^ T cells. Mice were euthanized and analyzed 28 days after transfer. Mice injected with αSLAMF3 had significantly bigger spleen size, weight and total number of splenocytes compared to isotype control (Figure S3). The levels of anti-chromatin, anti-single-stranded DNA (ssDNA) and double-stranded DNA (dsDNA) were substantially increased in the serum of mice that had received αSLAMF3 as compared to isotype control (Figures 2A–C). Percentage and numbers of marginal zone B cells decreased while total number of B cells in the spleen remained unchanged (Figure S3). Consistent with the increase in autoantibody production, the percentage of plasma cells was significantly expanded in the spleen of αSLAMF3-injected mice (Figure 2D). To further validate these observations, lymphoid follicles and GCs of recipient mice were measured with immunofluorescence staining from frozen spleen sections. B cell zones were identified by B220 and GC area was marked by GL7^+^ zone surrounded by IgD^+^ naive B cells. This showed that the size of Germinal Centers was increased in B6 recipients that had received two injections of anti-SLAMF3 (Figure 2E). Similar results were obtained with 4 injections of αSLAMF3 in bm12 CD4^+^ T cell transfers and αSLAMF1 was used as a control (see Figure S5). Anti-SLAMF3 injected group had significantly higher percentages of GC B cells and increased autoantibody production, whereas αSLAMF1 did not promote autoantibody production (Figures S5, S6).

### Anti-SLAMF3 Accelerates GC B Cell Differentiation Initiated by cGVHD

**Figure 3:**
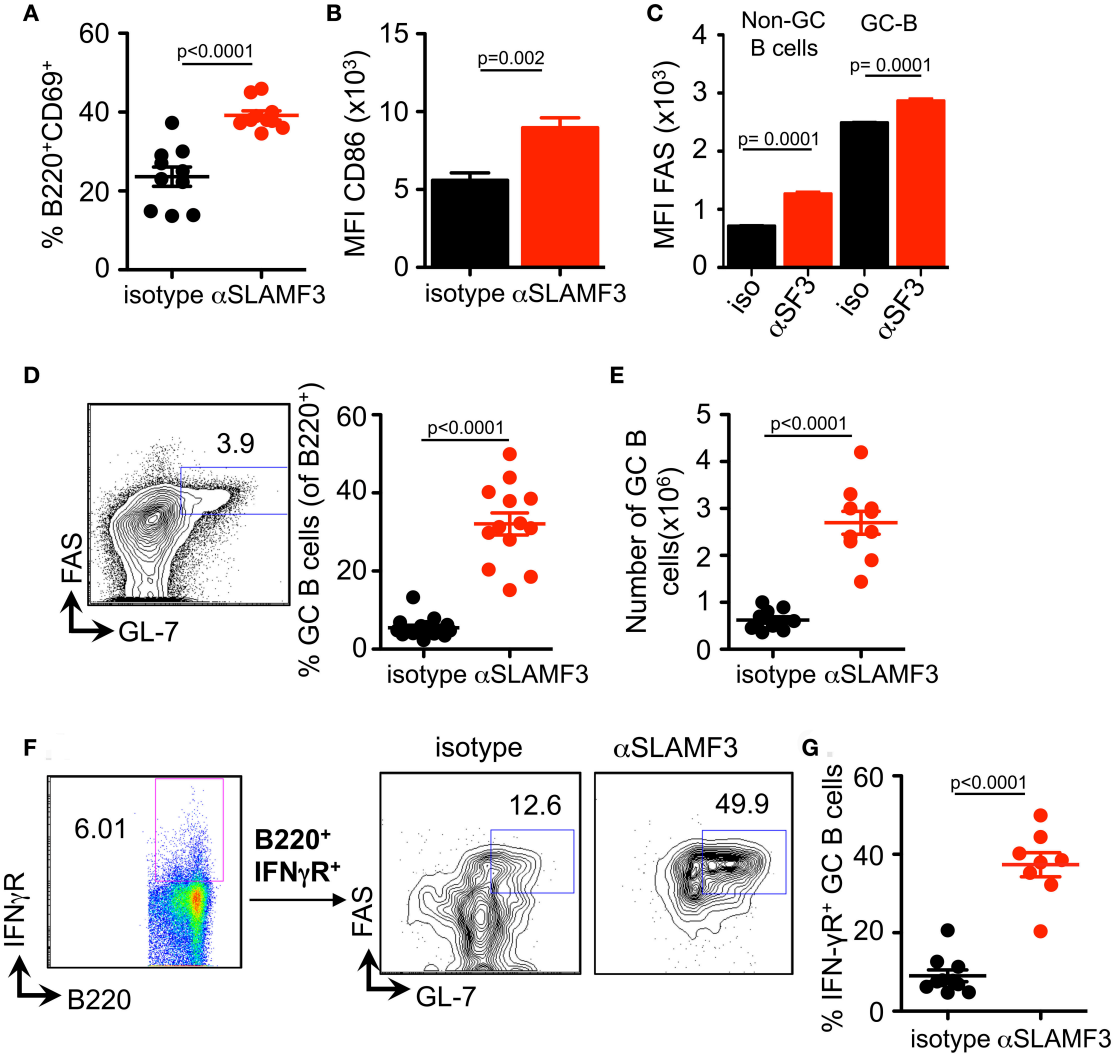
Anti-SLAMF3 accelerates GC B cell differentiation initiated by cGVHD. Anti-SLAMF3 or isotype injected bm12 CD4^+^ T cell transferred WT recipient mice were analyzed on day 14 when GC reactions peaked. **(A)** Percentages of B220^+^CD69^+^ B cells are as shown. **(B)** Expression of CD86 on B cells. **(C)** FAS expression in non-GC B and GC B cells from isotype and aSLAMF3 injected recipients. **(D,E)** Representative Flow cytometry plot for GC staining: CD19+GL-7+FAS+ B cells (left panel), percentages of GC B (Right panel) and numbers of GC B cells in spleen of mice. **(F)** Representative FACS plots of B220^+^IFN-γR^+^ GC B cells from isotype and αSLAMF3-injected recipients. **(G)** The percentage of B220^+^IFN-γR^+^ GC B cells in the spleens of isotype and αSLAMF3-injected recipients. Data represent at least four independent experiments.

Selection, isotype switching and expansion of GC B cells require critical signals from T follicular helper (TFH) cells. During chronic GVHD autoantibodies are produced due to the generation of autoimmune TFH and GC B cells in response to the donor CD4^+^ T cells (9). To assess whether the increased autoantibody production in mice injected with αSLAMF3 was the consequence of modulating the GC reaction, we first examined the presence of GC B cells in the spleen of recipient mice, 14 days post transfer of bm12 CD4^+^ T cells. In keeping with enhanced GC formation, the expression of CD86 and the proportion of CD69 activated B cells were markedly increased in anti-SLAMF3 injected recipients (Figures 3A,B). Surprisingly, the expression of FAS was not only increased on the surface of GC B cells, but also on all B cells (Figure 3C). As expected, flow cytometry analyses revealed a significant expansion of the percentage and number of CD19^+^FAS^+^GL-7^+^ GC B cells in αSLAMF3-injected recipients as compared to isotype control (Figures 3D,E).

Previous studies have indicated a key role for the IFN-γ receptor (IFN-γR) in development of autoantibody production in lupus-prone mice, e.g., MRL/Lpr, NZB/W, B6.Sle1b, and Roquin san/san (15–18). Deletion of IFN-γR on B cells abrogates formation of autoimmune GCs and autoimmunity (19). Based on these observations, we examined the surface expression of IFN-γR on GC B cells from recipients of bm12 CD4^+^ T cells. In parallel with the enhanced GC formation, administration of αSLAMF3 markedly increased percentage of IFN-γR^+^ GC B cells, compared to recipients that received isotype control (Figures 3F,G). These findings suggest that SLAMF3 indeed enhances the expansion of autoreactive GC B cells and that the αSLAMF3 antibody positively affects this cGVHD driven-expansion.

### Administering αSLAMF3 Increases T Cell Activation and TFH Cell Differentiation Initiated by cGVHD

**Figure 4:**
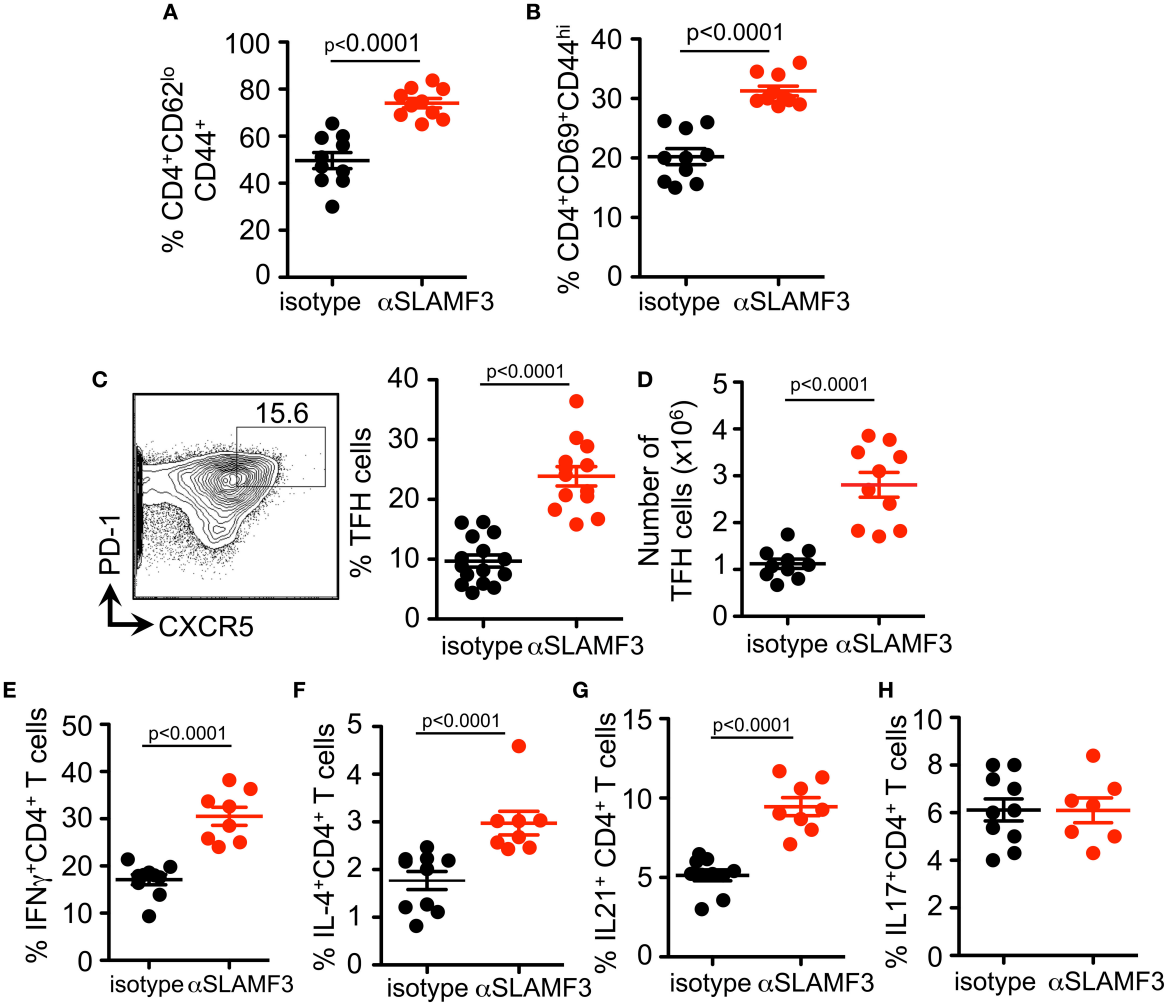
Administering αSLAMF3 increases T cell activation and TFH cell differentiation initiated by cGVHD. Anti-SLAMF3 or isotype injected bm12 CD4+ T cell transferred B6 WT recipient mice were analyzed on day 14 when GC reactions peaked. **(A,B)** Percentages of CD4^+^CD44^+^CD62^lo^ memory T cells and CD4^+^CD69^+^CD44^hi^ effector T cells in isotype and αSLAMF3 injected mice. **(C)** Representative Flow cytometry plot showing gating strategy for TFH cells: CD4^+^PD-1^+^CXCR5^+^ (left panel) Percentages of TFH cells in spleen of αSLAMF3 and isotype injected mice (right panel). **(D)** Absolute numbers of TFH cells in isotype and aSLAMF3 injected mice. **(E,H)** Expression of following cytokines was measured by intracellular staining in CD4^+^CD45.1^+^ splenocytes from isotype and αSLAMF3-injected recipients. Percentages of CD4^+^IFN-γ^+^ T cells, CD4^+^IL4^+^ T cells, CD4^+^ IL-21^+^ T cells, and CD4^+^ IL-17^+^ T cells are shown.

As the cGVHD in B6 recipients is initiated and driven by the transfer of bm12 co-isogenic CD4^+^ T cells we further analyzed the T cell compartment. As judged by expression of CD44, CD62, and CD69, the percentage and number of effector CD4^+^ T cell were also higher in αSLAMF3 treated recipients (Figures 4A,B). In accordance with the increase in total CD4^+^ T cells, the percentage of CD4^+^CXCR5^+^PD-1^+^ TFH cells were significantly increased in αSLAMF3-injected recipient mice (Figure 4C). PD1 expression was not only increased on the surface of CD4 of TFH cells, we also found an increase in number and expression of PD1^+^ CD4^+^ T cells in αSLAMF3-injected recipients (Figure S4). Importantly, these activated CD4^+^ T cells did not only secrete more IFNγ in response to administering, but also increased amounts of the key cytokines IL-4 and IL-21, while IL-17 remained unchanged (Figures 4E–H). These findings correlate with reports that SLE patients as well as lupus-prone mice have increased serum levels of IL-21, IFNγ, and IL-17 (20–23). We conclude that the high levels of IL-4, IL-21, and IFNγ in cGVHD are further enhanced by triggering αSLAMF3, which drives the dysregulated GC reaction.

### Selective Increase of the Number of Donor CD4^+^CD45.1^+^ T Cells Upon Administering αSLAMF3 During cGVHD

**Figure 5:**
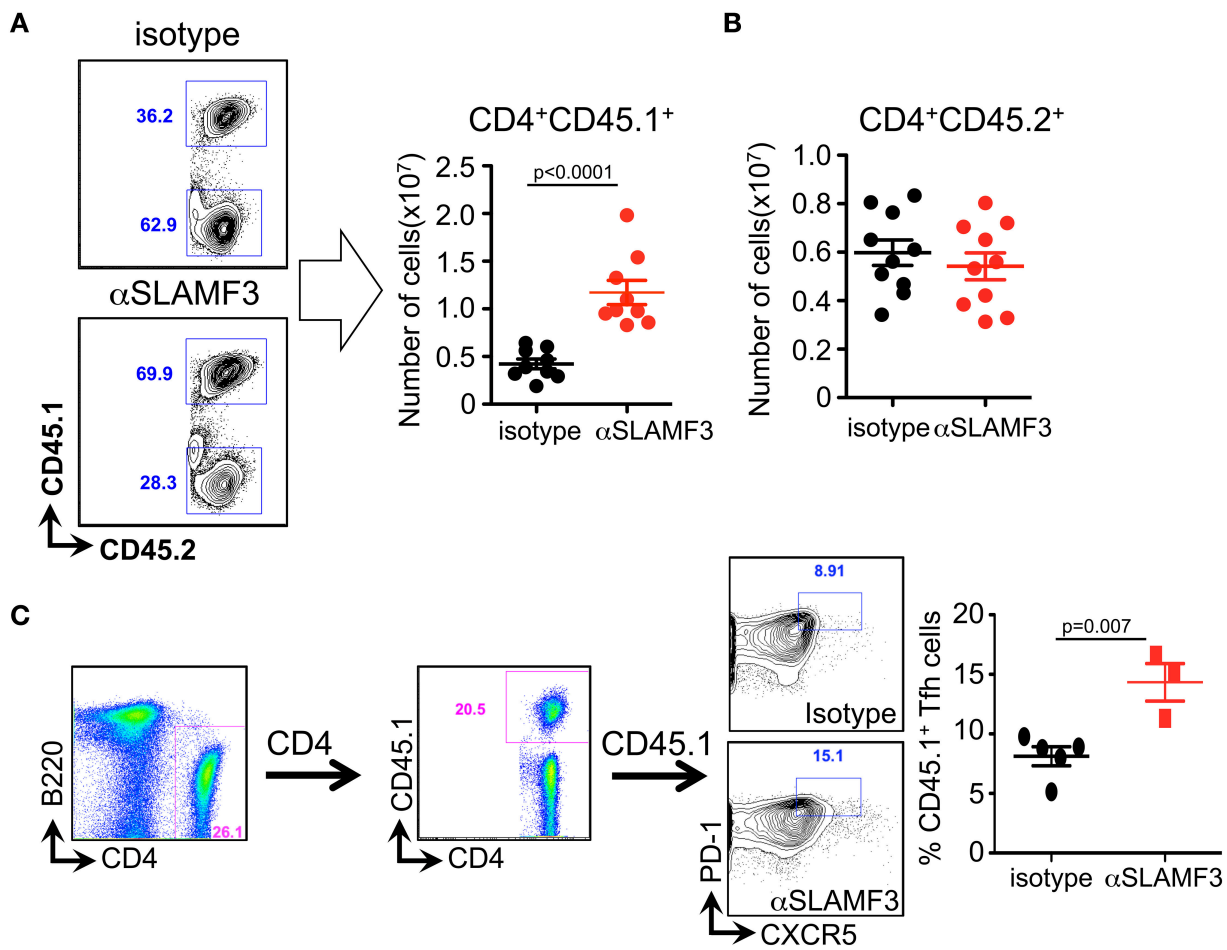
Selective increase of the number of donor CD4^+^CD45.1^+^ T cells upon administering αSLAMF3 during cGVHD. **(A)** Representative dot plots of CD4^+^CD45.1^+^ T cells from isotype and αSLAMF3-injected recipients (left panel). The number of CD4^+^CD45.1^+^ donor T cells in the spleens of isotype and αSLAMF3-injected recipients (right panel). **(B)** The number of CD4^+^CD45.2^+^ recipient T cells in the spleens of isotype and αSLAMF3-injected recipients. **(C)** Representative FACS plots of CD45.1^+^ donor TFH cells (left) with percentages of CD45.1^+^ TFH cells in isotype and αSLAMF3 (right). Data represent at least four independent experiments.

In order to distinguish between the role of donor and recipient T cells in the GC reaction, we crossed bm12 and CD45.1 mice and subsequently transferred bm12xCD45.1 donor CD4^+^ T cells into recipient CD45.2^+^ B6 WT mice. Fourteen days after transfer, mice were euthanized and CD4^+^ T cells were analyzed. Significantly, only the donor CD4^+^ T cells (CD4^+^CD45.1^+^) had expanded in αSLAMF3-injected mice, while recipient CD4^+^ T cells were unaffected (Figures 5A,B). More specifically, we identified significantly expanded CD45.1^+^ donor TFH cells in the recipient mice injected with αSLAMF3 (Figure 5C).

### Upon Induction of Chronic GVHD by the Transfer of bm12 CD4^+^ Cells Into B6 Mice Donor Cell Treg and TFR Development Is Selectively Impaired by αSLAMF3

**Figure 6:**
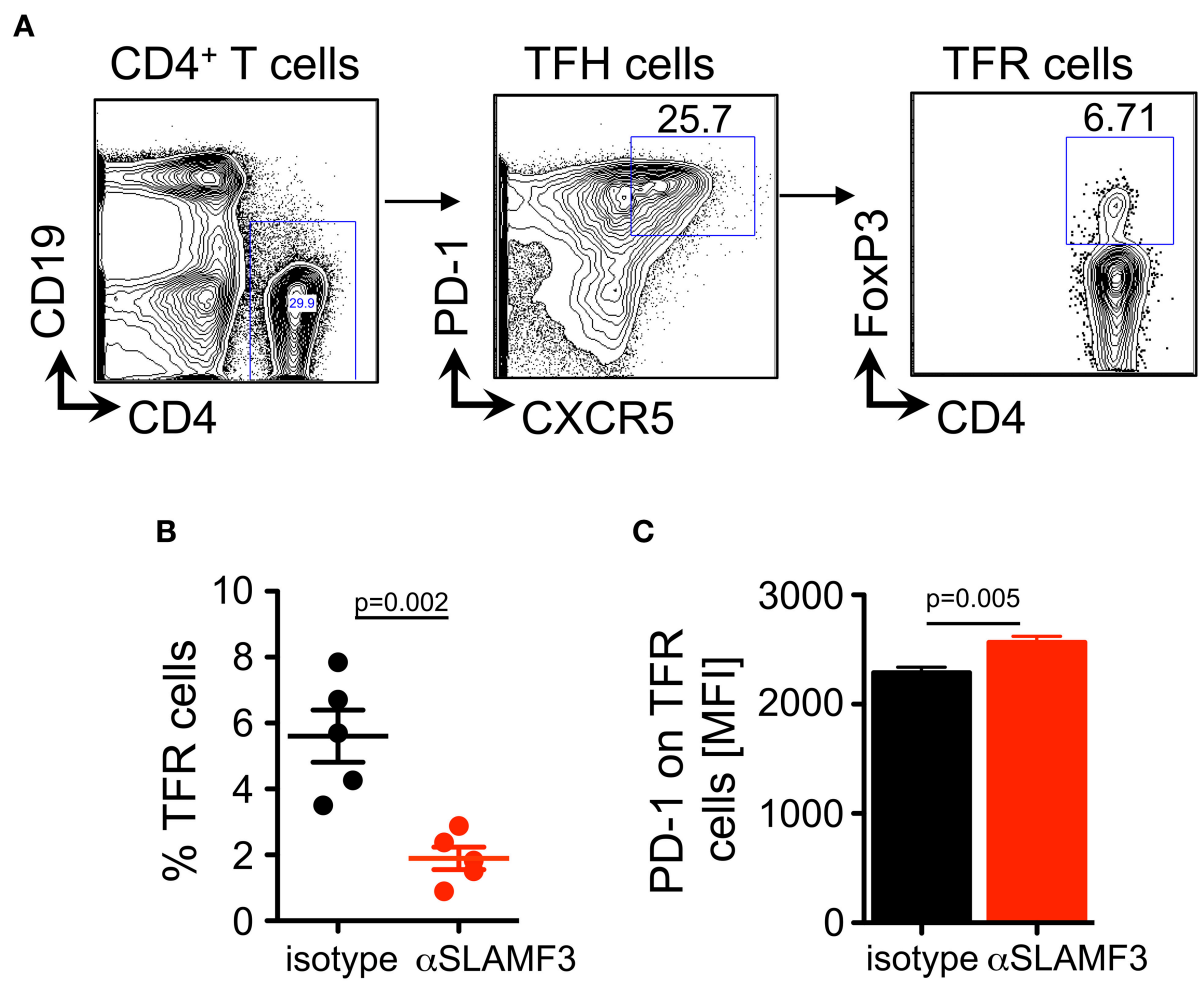
Anti-SLAMF3 inhibits TFR cell differentiation. **(A)** Representative gating strategy of TFR cells from spleens of the recipients of bm12 CD4+ T cells: CD4^+^CXCR5^+^PD-1^+^Foxp3^+^ **(B)** Percentages of TFR cells in the spleens of isotype and αSLAMF3-injected mice. **(C)** Cell surface expression of PD-1 on TFR cells in the spleens of isotype and αSLAMF3-injected mice. Data represent at least four independent experiments.

Regulatory T (Treg) cells have been demonstrated to play vital roles in suppressing cellular and humoral immune responses, i.e., by suppressing autoreactive B cell functions and subsequent autoantibody production (24–26). Of interest, recent studies have identified a subset of regulatory T cells in the GCs, termed T follicular regulatory (TFR) cells that suppress TFH and GC B cells (27). Therefore, we hypothesized that TFR cells may be affected from injections of αSLAMF3. Fourteen days after transfer of bm12 CD4^+^ T cells into B6 WT recipients and subsequent 2 injections of αSLAMF3, mice were euthanized and analyzed for TFR cells at the peak of GC reactions. TFR cells, defined as CD4^+^CXCR5^+^PD-1^+^FoxP3^+^ were significantly reduced from spleen of αSLAMF3-injected mice as compared to isotype control (Figures 6A,B). Furthermore, TFRs present in αSLAMF3-injected mice had significantly higher PD1 expression compared to isotype control (Figure 6C). This suggests that negative regulation within the GCs is lost upon αSLAMF3 injections.

**Figure 7:**
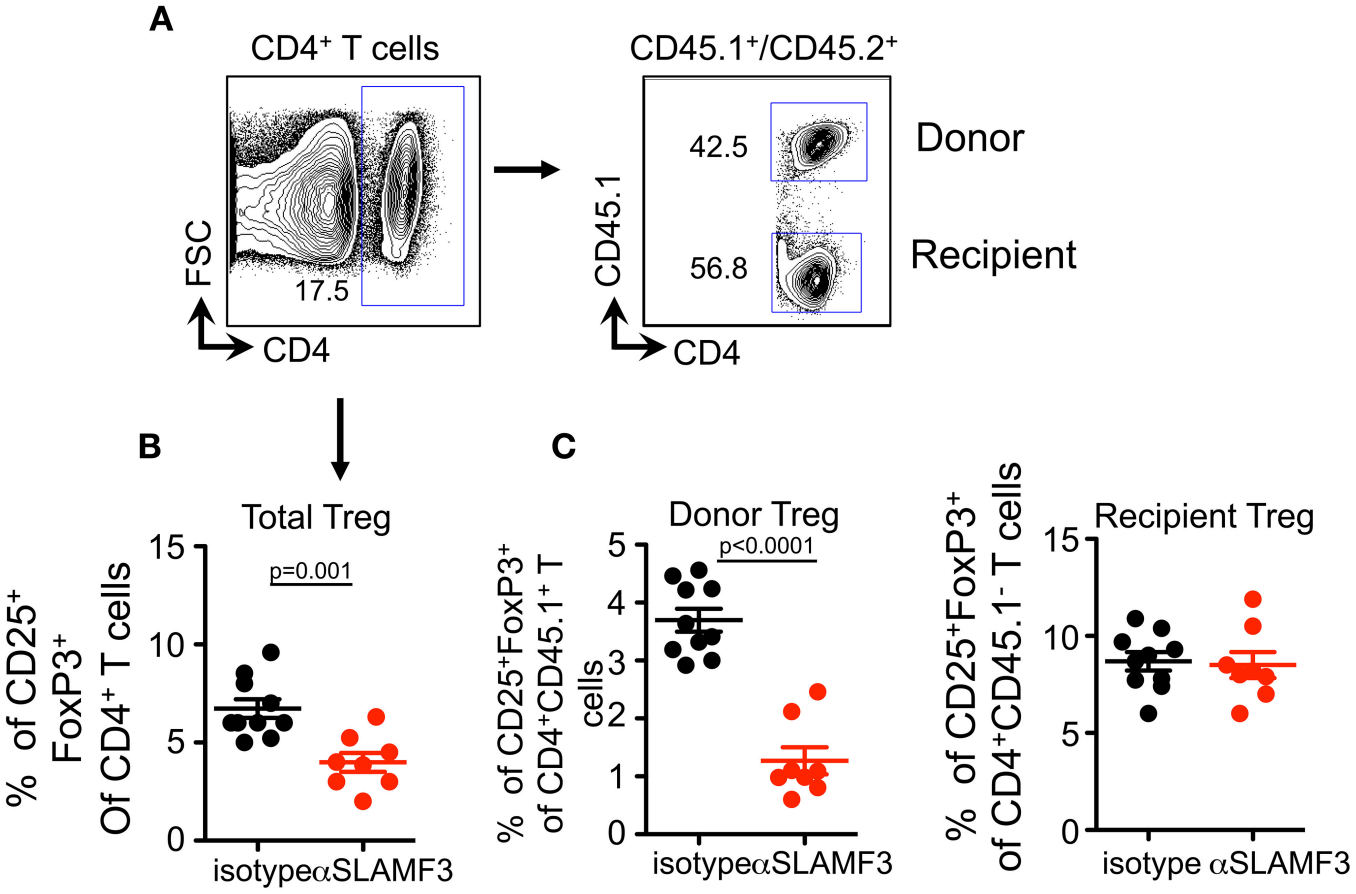
Upon induction of chronic GVHD by the transfer of bm12 CD4^+^ T cells into B6 mice donor cell Treg development is selectively impaired by αSLAMF3. **(A)** Representative FACS plots of total CD4^+^ T and subsequent donor/recipient CD4^+^CD45.1^+^ and CD4^+^CD45.2^+^, respectively. Of these subsets, Treg population was determined as CD25^+^FoxP3^+^. **(B)** Percentage of total CD4^+^CD25^+^Fox3^+^Treg cells in the spleens of isotype and αSLAMF3-injected recipients. **(C)** Percentages of CD4^+^CD45.1^+^CD25^+^Fox3^+^ donor Treg (left panel) and CD4^+^CD45.1^−^CD25^+^Fox3^+^ recipient Treg (right panel) cells in the spleens of isotype and αSLAMF3-injected recipients.

As TFR cells originate from Treg precursors (27), we checked whether SLAMF3 signaling also has negative effect on Treg differentiation. Similar to the impact on TFR cells, αSLAMF3-injected recipients exhibited a substantial decrease in the frequency of CD4^+^Foxp3^+^CD25^+^ T cells compared with isotype control injected mice (Figures 7A,B). Strikingly, the impact of anti-SLAMF3 was specific to donor-derived Treg cells because the administration of αSLAMF3 exerted no effect on recipient-derived Treg cells (Figure 7C). Together, these data demonstrate that SLAMF3 signaling not only inhibited TFR cell differentiation but also of Treg cells, which may contribute to exacerbated autoimmune responses in αSLAMF3-injected recipients.

## Discussion

In studies of murine autoimmune-like chronic graft vs.-host disease (cGVHD), B cells are activated by donor CD4^+^ T cells to upregulate MHC II and costimulatory molecules. Acting as efficient APCs, donor B cells further augment donor the clonal expansion, differentiation, and survival of pathogenic CD4^+^ T cells, which drives autoreactivity. Because SLAMF3-deficient mice develop autoantibodies irrespective of their genetic background, and as development several B cell subsets, e.g., Marginal zone [MZ] B cells are eliminated in these mice, we adopted a cGVHD model to investigate the importance of SLAMF3 signaling in this disease. To this end we examined the effect of a mouse anti-mouseSLAMF3 monoclonal antibody on hapten-induced humoral responses and on autoantibody production in the bm12 CD4^+^ > B6 transfer model of cGVHD.

The latter model was chosen because it mirrors a few common pathways of human disease, e.g., high levels of circulating anti-nuclear antibodies, concomitantly with large frequencies of T follicular helper cells (Tfh), germinal center (GC) B cells, and plasma cells. We previously had shown that by using lupus—prone B6 mutants in this cGVHD model the autoantibody production is accelerated. Conversely, specific monoclonal antibodies, which affect GC formation in mice that are immunized with foreign antigens, also ameliorate the generation of anti-nuclear antibodies in this bm12>B6 cGVHD model (11, 12). As also shown by others, cGVHD can be induced both by the transfer of bm12 derived CD4^+^ cells into B6 mice or by B6-derived CD4^+^ cells into the co-isogenic bm12 recipients with equal efficiency (8, 9).

In our analyses of NP-OVA immunizations combines with administering αSLAMF3, we demonstrate that in response to foreign antigens, SLAMF3 primarily affects the maturation into GC B cells, leading to diminished antibody responses. By contrast, injections of αSLAMF6 in NP-OVA immunized mice, SLAMF6 signaling affected both B and T cells in the germinal centers (2).

Precise regulation of TFH cell numbers is critical for optimal humoral responses, and aberrant expansion of TFH cells is associated with autoimmune diseases, including lupus (28, 29). In our system, we found that the administration of αSLAMF3 significantly enhanced proliferation of donor-derived CD4^+^ T cells, which lead to profound TFH cell differentiation in GCs. As a consequence, we observed severe autoimmune phenotypes in αSLAMF3-injected recipients, which included increased serum levels of anti-dsDNA, anti-ssDNA, and anti-chromatin autoantibodies (Figure 1). This was a consequence of enlarged germinal centers with massive accumulation of GC B cells in the spleens (Figure 2). Although several molecules, including CD40L, ICOS, PD-1, and SAP, are known to be involved in TFH cell differentiation, the transcriptional repressor Bcl6 is found to be a lineage-defining factor for TFH cells. Bcl6 is necessary to specify the TFH cell program and overexpression of Bcl6 is sufficient to drive TFH cell differentiation (30). Indeed, the expression of Bcl6 was markedly increased in αSLAMF3-injected recipients, but not in isotype control injected recipient mice (Unpublished observation). Beside BCR signaling, the survival and selection of GC B cells within GCs are dependent on survival signals from GC TFH cells. The experiments reported here are consistent with strong TFH cell differentiation with extensive GC B cell responses. Recent publications have suggested that B cells have a cell-intrinsic requirement for expression of CD80 and/or CD86 for differentiation into GC B cells (31, 32). In accord, we found increased expression of CD86 in B cells and GC B cells in αSLAMF3-injected recipient mice.

Interestingly, IFN-γR signals are shown to synergize with BCR, TLR, and CD40 dependent signal to enhance expression of the GC master regulator transcription factor Bcl6 (19), which suggest IFN-γ facilitates autoimmune GC formation by initiating a GC transcriptional program. Based on these findings by others, we examined the expression Bcl6 and IFN-γR and found that GC B cells exhibited increased Bcl6 and IFN-γR. In addition, we observed higher expression of anti-apoptotic protein Bcl-2 in αSLAMF3-injected GC B cells (unpublished observation). This in turn may reduce overall apoptosis of GC B cells and ultimately result in increased numbers of GC B cells in the GCs. It will be of interest to determine whether the expression of other members of the Bcl-2 family, such as Mcl-1, are also increased in αSLAMF3-injected GC B cells. Thus, it appears that αSLAMF3 may act on both B and T cell sides during autoimmune GC reactions.

In addition, αSLAMF3 had a pronounced effect on key cytokines produced during GC reactions. IL-21, IL-4, and IFN-γ production were significantly elevated in donor-derived CD4^+^ T cells of αSLAMF3-injected recipients. Although IL-21 production is restricted to activated CD4 T and NKT cells, IL-21 receptor is expressed on a variety of immune cells (33, 34). IL-21 promotes the differentiation and expansion of TFH cells, regulates B cell proliferation and survival, GC formation, and plasma cell differentiation (35–37). Furthermore, IL-21 has an important role in regulating T cell-dependent B cell responses, partly in cooperation with IL-4 (38). IL-21 is markedly elevated in autoimmune-prone mice and lupus severity is diminished in the absence of IL-21 or IL-21R signaling (21, 39). Besides IL-21, IFN-γ also contributes to lupus in both human and murine models. The increased serum IFN-γ levels are associated with disease activity and the inhibition of IFN-γ expression prevents the development of murine lupus (40, 41). Thus, the combined alteration of IL-21, IFN-γ, and IL-4 production in αSLAMF3-injected recipients may play an important role in higher GC formation and autoantibody production.

We were surprised to find that αSLAMF3 injections resulted in reduced numbers of Treg cells in the recipients of bm12 CD4^+^ T cells. The pivotal roles of Treg cells in the development and maintenance of immune self-tolerance have been well documented (42). It was thought that Treg cells functioned as immuno-suppressors in allogeneic immune responses and in autoimmune responses. In this study, recipient Treg cells does not seem to play a major role in suppressing lupus-like phenotype as similar numbers of Treg cells were differentiated in both αSLAMF3 and isotype-injected recipient mice. In contrast, donor-derived Treg cells were significantly reduced after αSLAMF3 injections. Thus, these data suggest that αSLAMF3 specifically limits proliferation of donor-derived Treg cells, but have minimal effect on proliferation of host Treg cells. Perhaps the most striking finding in our studies was the reduced differentiation of donor-derived TFR cells. TFR cells are another subset of CD4^+^ T cells in the GCs (43, 44). TFR cells share phenotypic characteristics with TFH but are derived from suppressive FoxP3^+^ Treg cells. Within the GC, TFR cells inhibit GC formation and restrict the autoimmune responses (45). Our findings indicate that the reduced number of TFR cells that leads to reduced suppression on TFH cells in the GCs leads to increased autoantibody production in αSLAMF3-injected recipients. Although the suppressive capacity of TFR cells is not examined, the higher expression of PD-1 on TFR cells indirectly suggest there may be reduced suppressive functions in αSLAMF3-injected recipients. Further work is needed to understand how PD-1 expression is regulated *in vivo* by SLAMF3 signaling. However, the increased expression of PD-1 on TFR cells might partly contribute to the reduced numbers of TFR cells in αSLAMF3-injected recipients. This idea is supported by previous observations that mice deficient in PD-1 have increased numbers of TFR cells with enhanced suppressive capacity (46). Collectively, impaired Treg and TFR compartments could enhance TFH activity, resulting in the expansion of autoreactive B cells and autoantibody production.

In the cGVHD model, the key cellular mechanism that results in the loss of B cell tolerance is the interaction of donor CD4^+^ T cells with MHC class II on host B cell surface. During T-B cell interactions, allogeneic donor CD4^+^ T cells provide the abnormal T cell help to host B cells that appears to have functional consequences different from what occurs in spontaneous model. Our results suggested that αSLAMF3 influences the development of cGVHD through regulating TFR/Treg cell ratios, as agonist αSLAMF3 induces hyperactivation of activated donor CD4^+^ T cells. However, how αSLAMF3 has a pronounced effect on the donor T differentiation remains unclear. Donor T cells have classically been considered the main effector cells, but host APCs cross-present host auto-antigens to donor T cells, which will be activated and start proliferating. Because SLAMF3 is expressed on both T cells and APCs, triggering the receptor on either on T cells or APCs may induce co-stimulatory signaling, Although Treg cell adoptive therapy is showing potential as a treatment of cGVHD patients (47), generating sufficient Treg cells remains a challenge.

In summary, the dual activity of SLAMF3 signaling as inhibitory or activating, depending on the setting of the immune response, suggests that SLAMF3 should be further investigated as a target in a disease dependent manner in humans.

## Ethics Statement

All animals are maintained under specific pathogen-free conditions at the Beth Israel Deaconess Medical Center (BIDMC) animal facility. Experiments were performed according to the guidelines of the Institutional Animal Care and Use Committee (IACUC) at BIDMC.

## Author Contributions

NW, BY, and CvdP designed and did the experiments. NW, BY, and CT wrote the manuscript. MC, MCC, RH, PE, and CT were involved in discussion of the results. All authors read and approved the manuscript.

### Conflict of Interest Statement

The authors declare that the research was conducted in the absence of any commercial or financial relationships that could be construed as a potential conflict of interest.
