# Supplementary material for: The Checkpoint Regulator SLAMF3 Preferentially Prevents Expansion of Auto-Reactive B Cells Generated by Graft-vs.-Host Disease
Source: Front Immunol. 2019 Apr 17;10:831. doi: 10.3389/fimmu.2019.00831 (PMC6482334; doi:10.3389/fimmu.2019.00831)
Supplement: Supplementary file 1 [file Presentation_1.PPTX]

## Slide 1
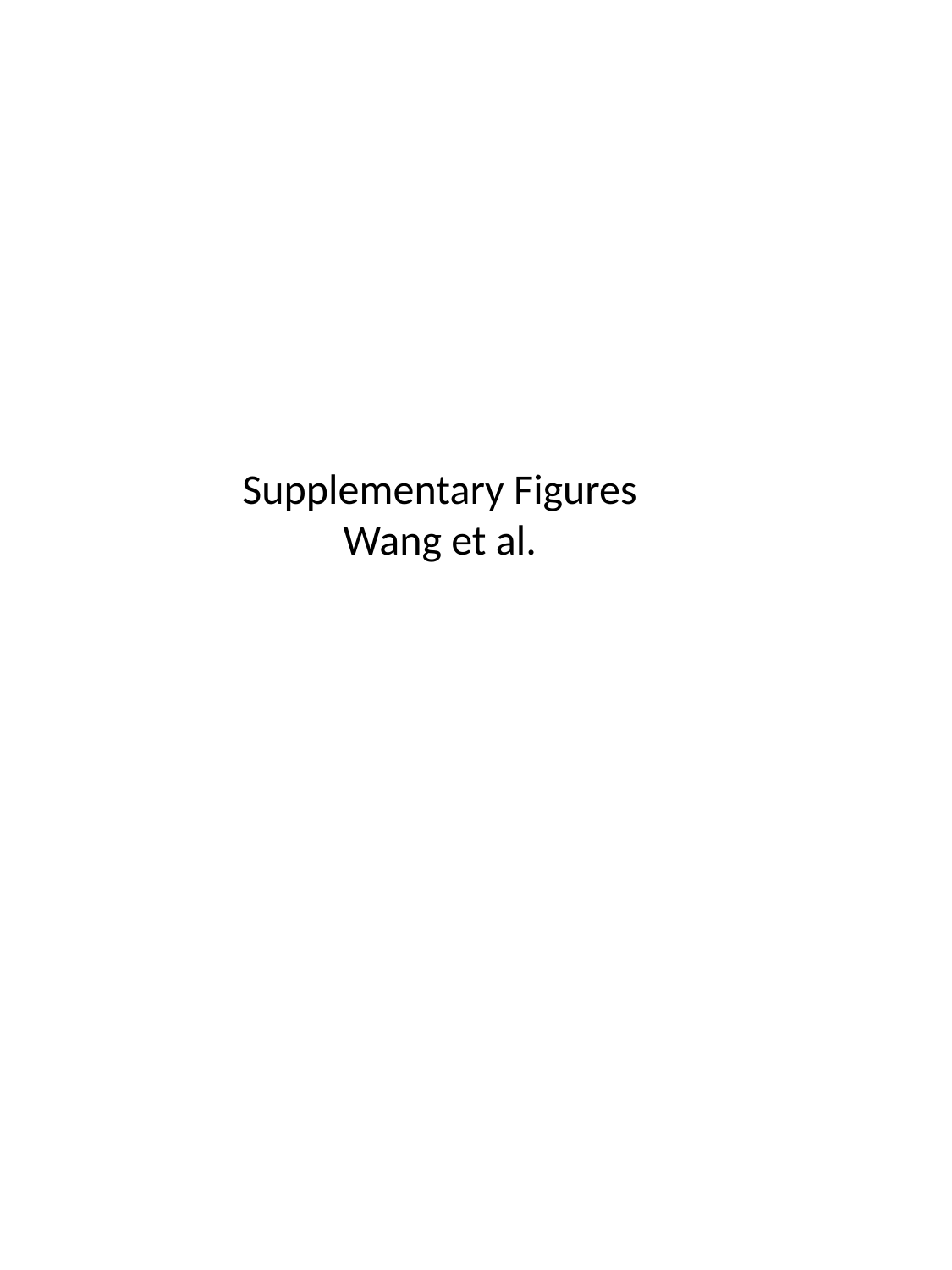

Supplementary Figures
Wang et al.

## Slide 2
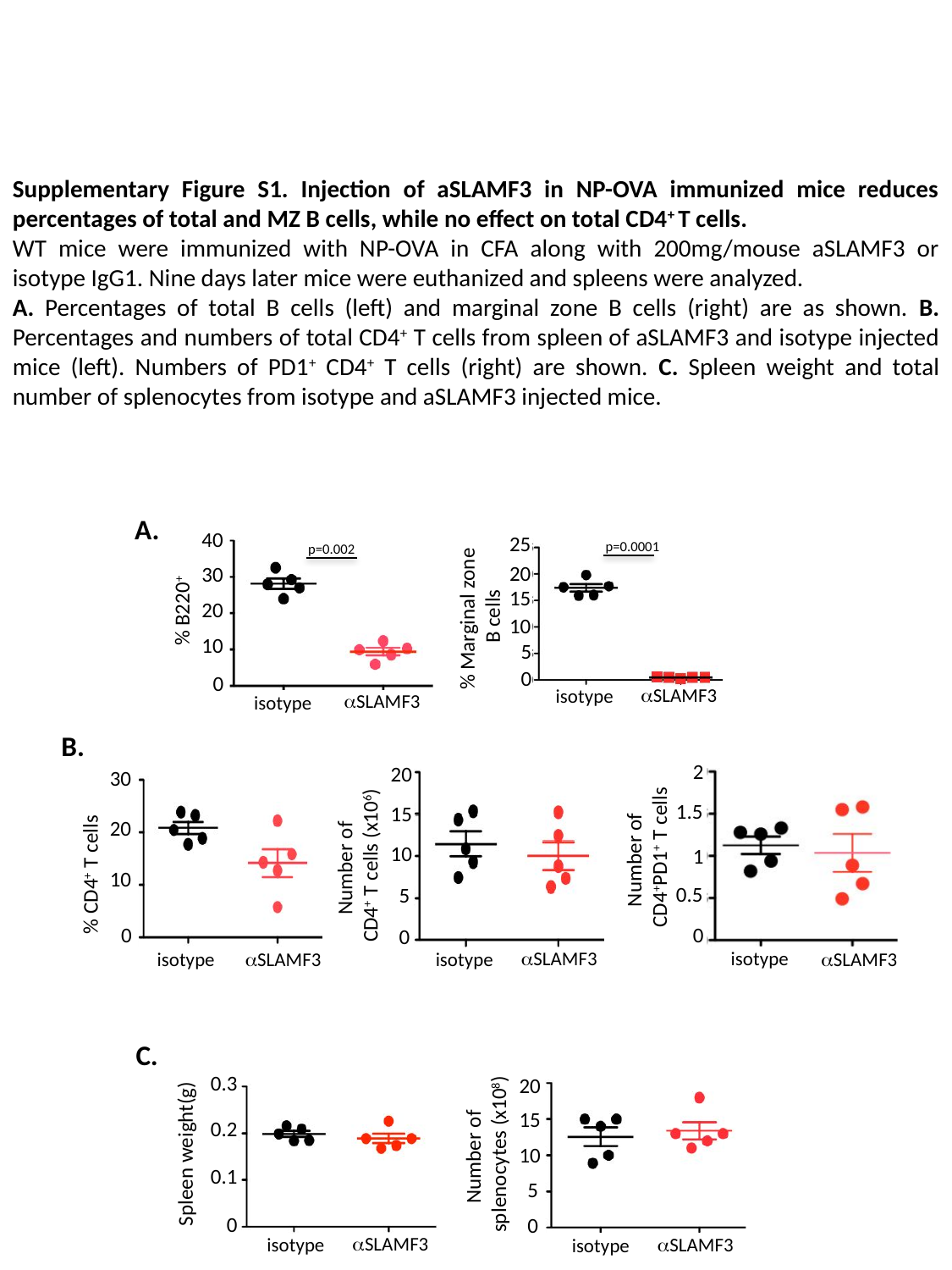

Supplementary Figure S1. Injection of aSLAMF3 in NP-OVA immunized mice reduces percentages of total and MZ B cells, while no effect on total CD4+ T cells.
WT mice were immunized with NP-OVA in CFA along with 200mg/mouse aSLAMF3 or isotype IgG1. Nine days later mice were euthanized and spleens were analyzed.
A. Percentages of total B cells (left) and marginal zone B cells (right) are as shown. B. Percentages and numbers of total CD4+ T cells from spleen of aSLAMF3 and isotype injected mice (left). Numbers of PD1+ CD4+ T cells (right) are shown. C. Spleen weight and total number of splenocytes from isotype and aSLAMF3 injected mice.
A.
40
p=0.002
30
20
% B220+
10
0
aSLAMF3
isotype
25
p=0.0001
20
15
% Marginal zone
B cells
10
5
0
aSLAMF3
isotype
B.
2
1.5
Number of
CD4+PD1+ T cells
1
0.5
0
isotype
 aSLAMF3
20
15
Number of
CD4+ T cells (x106)
10
5
0
aSLAMF3
isotype
30
20
% CD4+ T cells
10
0
isotype
 aSLAMF3
C.
20
15
Number of
splenocytes (x108)
10
5
0
aSLAMF3
isotype
0.3
0.2
Spleen weight(g)
0.1
0
aSLAMF3
isotype

## Slide 3
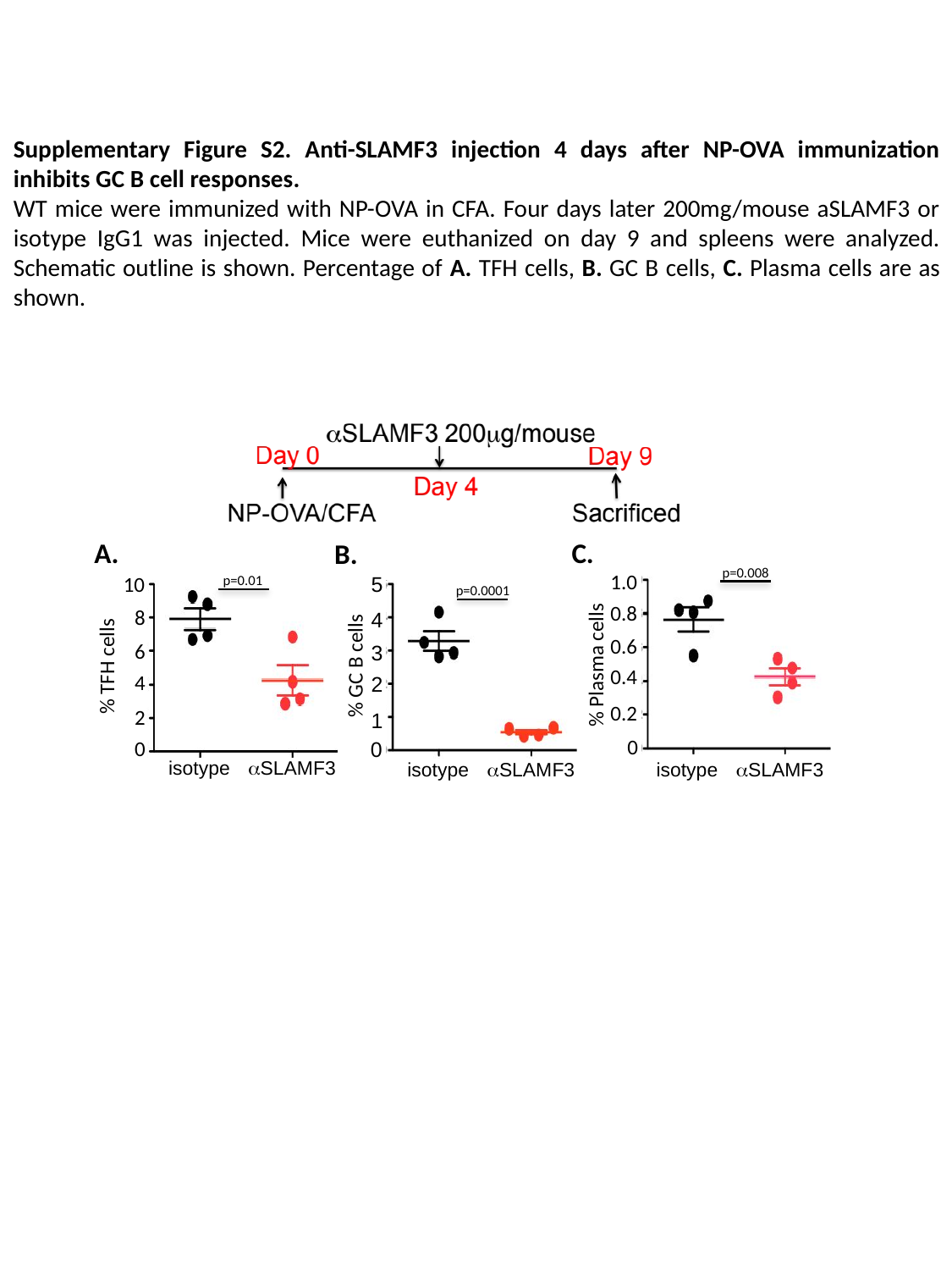

Supplementary Figure S2. Anti-SLAMF3 injection 4 days after NP-OVA immunization inhibits GC B cell responses.
WT mice were immunized with NP-OVA in CFA. Four days later 200mg/mouse aSLAMF3 or isotype IgG1 was injected. Mice were euthanized on day 9 and spleens were analyzed. Schematic outline is shown. Percentage of A. TFH cells, B. GC B cells, C. Plasma cells are as shown.
A.
C.
B.
p=0.008
1.0
0.8
0.6
% Plasma cells
0.4
0.2
0
isotype
aSLAMF3
10
p=0.01
8
6
% TFH cells
4
2
0
isotype
aSLAMF3
5
p=0.0001
4
3
% GC B cells
2
1
0
isotype
aSLAMF3

## Slide 4
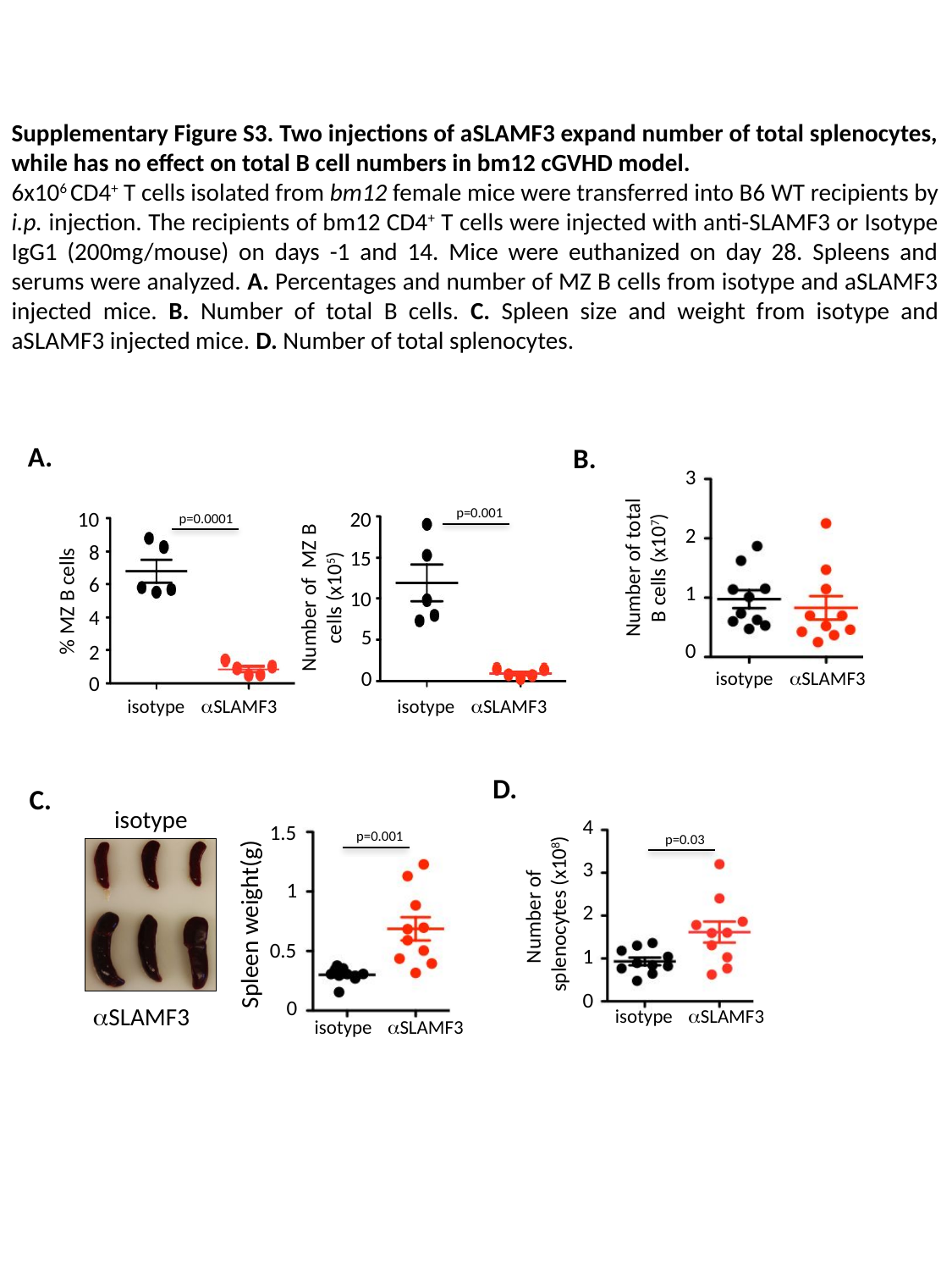

Supplementary Figure S3. Two injections of aSLAMF3 expand number of total splenocytes, while has no effect on total B cell numbers in bm12 cGVHD model.
6x10^6^ CD4+ T cells isolated from bm12 female mice were transferred into B6 WT recipients by i.p. injection. The recipients of bm12 CD4+ T cells were injected with anti-SLAMF3 or Isotype IgG1 (200mg/mouse) on days -1 and 14. Mice were euthanized on day 28. Spleens and serums were analyzed. A. Percentages and number of MZ B cells from isotype and aSLAMF3 injected mice. B. Number of total B cells. C. Spleen size and weight from isotype and aSLAMF3 injected mice. D. Number of total splenocytes.
A.
B.
3
2
Number of total
B cells (x107)
1
0
isotype
aSLAMF3
p=0.001
20
15
Number of MZ B cells (x105)
10
5
0
isotype
aSLAMF3
10
p=0.0001
8
6
% MZ B cells
4
2
0
isotype
aSLAMF3
D.
C.
isotype
aSLAMF3
1.5
p=0.001
1
Spleen weight(g)
0.5
0
isotype
 aSLAMF3
4
p=0.03
3
Number of
splenocytes (x108)
2
1
0
isotype
 aSLAMF3

## Slide 5
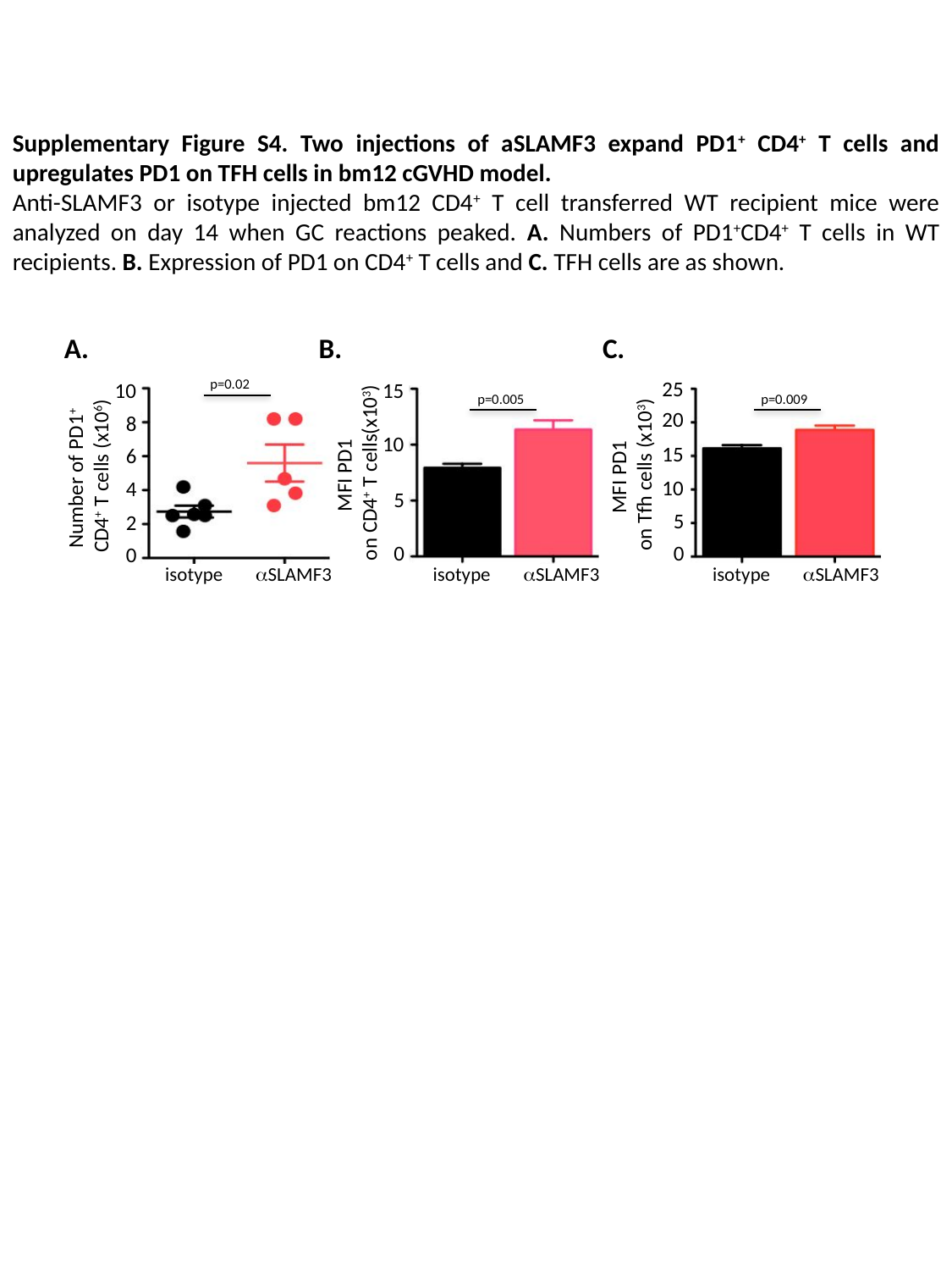

Supplementary Figure S4. Two injections of aSLAMF3 expand PD1+ CD4+ T cells and upregulates PD1 on TFH cells in bm12 cGVHD model.
Anti-SLAMF3 or isotype injected bm12 CD4+ T cell transferred WT recipient mice were analyzed on day 14 when GC reactions peaked. A. Numbers of PD1+CD4+ T cells in WT recipients. B. Expression of PD1 on CD4+ T cells and C. TFH cells are as shown.
A.
B.
C.
15
p=0.005
10
MFI PD1
on CD4+ T cells(x103)
5
0
isotype
 aSLAMF3
p=0.02
10
8
6
Number of PD1+
CD4+ T cells (x106)
4
2
0
isotype
 aSLAMF3
25
p=0.009
20
15
MFI PD1
on Tfh cells (x103)
10
5
0
isotype
 aSLAMF3

## Slide 6
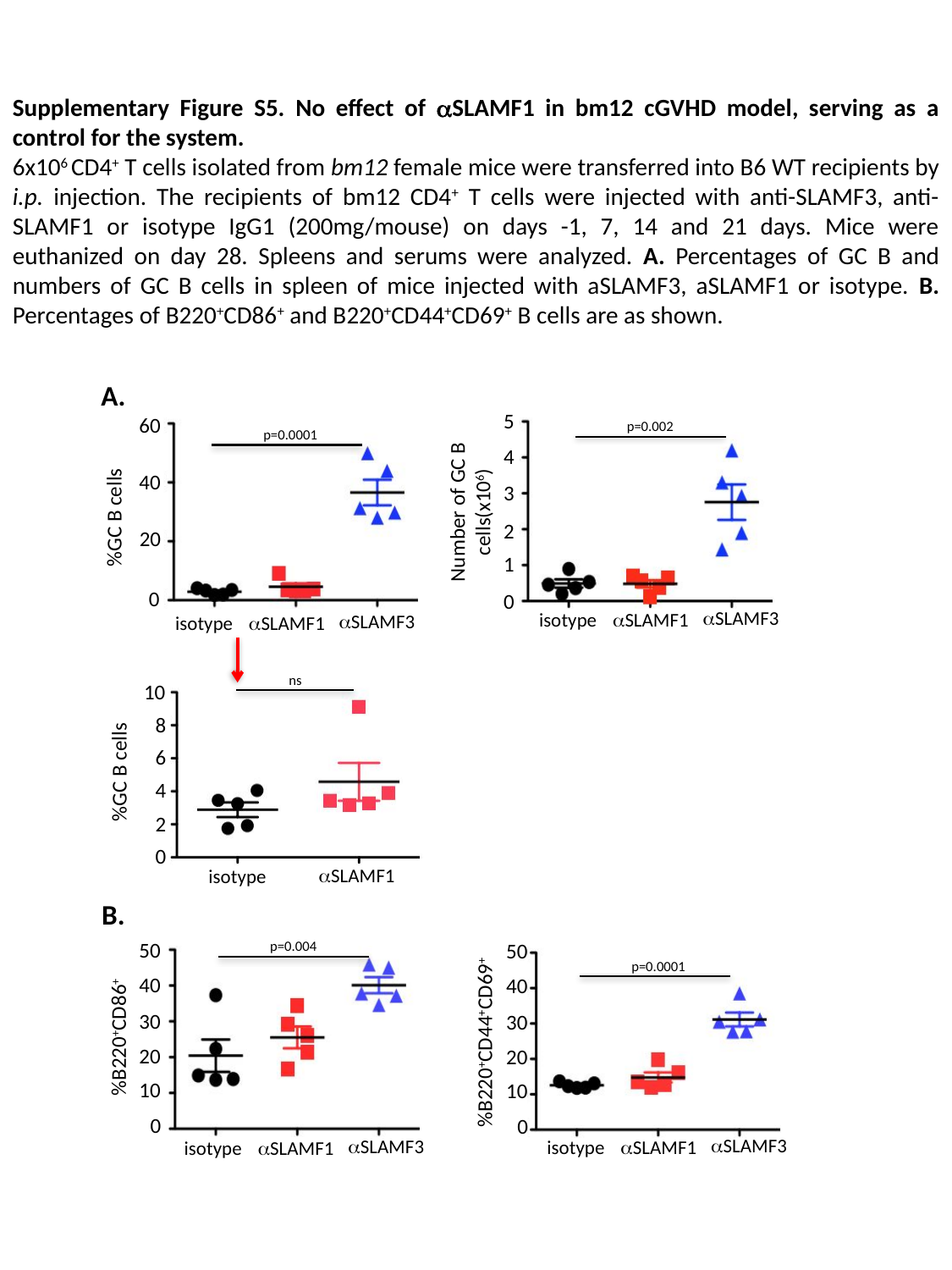

Supplementary Figure S5. No effect of aSLAMF1 in bm12 cGVHD model, serving as a control for the system.
6x10^6^ CD4+ T cells isolated from bm12 female mice were transferred into B6 WT recipients by i.p. injection. The recipients of bm12 CD4+ T cells were injected with anti-SLAMF3, anti-SLAMF1 or isotype IgG1 (200mg/mouse) on days -1, 7, 14 and 21 days. Mice were euthanized on day 28. Spleens and serums were analyzed. A. Percentages of GC B and numbers of GC B cells in spleen of mice injected with aSLAMF3, aSLAMF1 or isotype. B. Percentages of B220+CD86+ and B220+CD44+CD69+ B cells are as shown.
A.
5
p=0.002
4
3
Number of GC B cells(x106)
2
1
0
aSLAMF3
isotype
aSLAMF1
60
p=0.0001
40
%GC B cells
20
0
aSLAMF3
isotype
aSLAMF1
ns
10
8
6
%GC B cells
4
2
0
aSLAMF1
isotype
B.
50
p=0.0001
40
30
%B220+CD44+CD69+
20
10
0
aSLAMF3
isotype
aSLAMF1
50
p=0.004
40
30
%B220+CD86+
20
10
0
aSLAMF3
isotype
aSLAMF1

## Slide 7
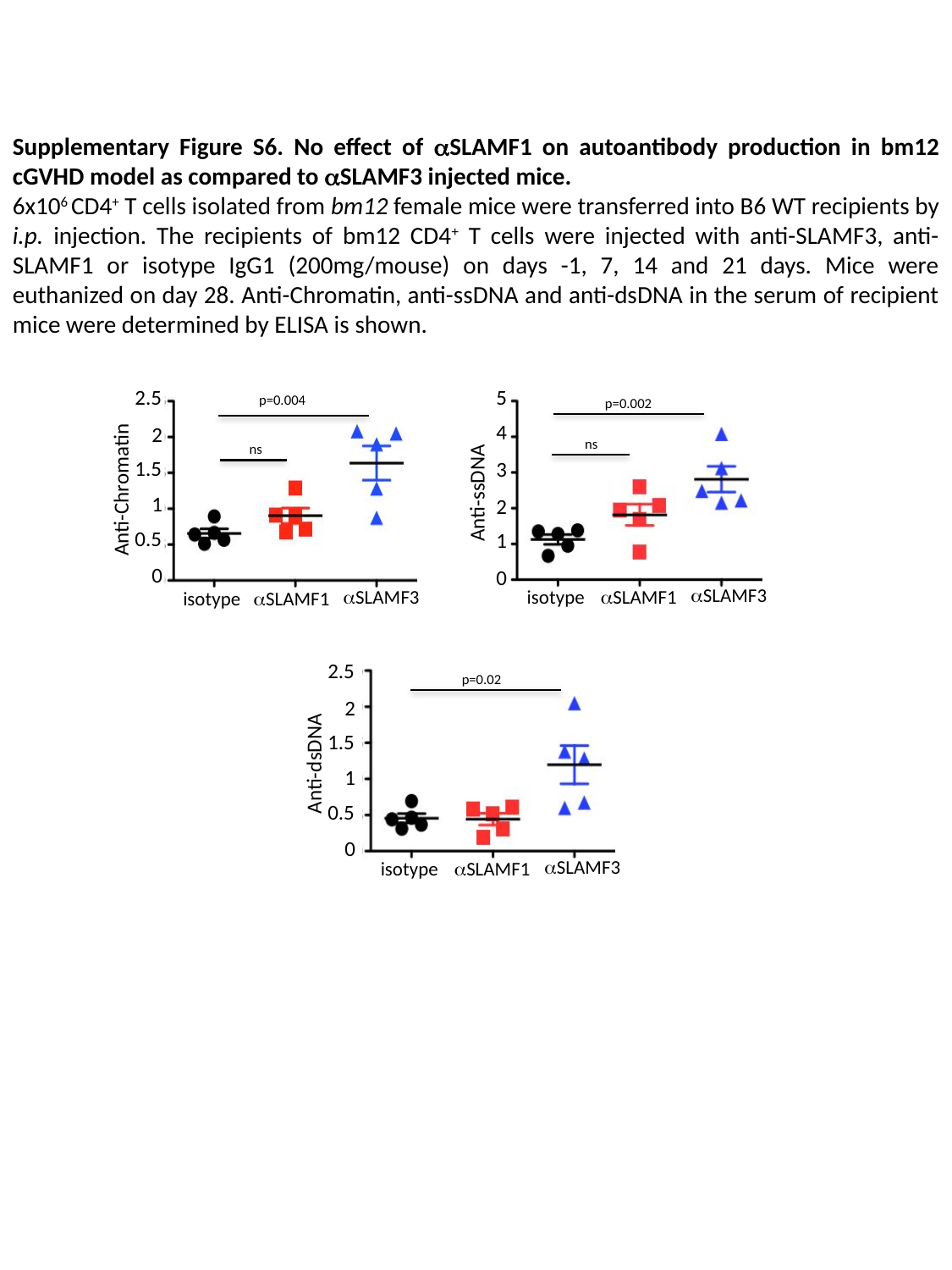

Supplementary Figure S6. No effect of aSLAMF1 on autoantibody production in bm12 cGVHD model as compared to aSLAMF3 injected mice.
6x10^6^ CD4+ T cells isolated from bm12 female mice were transferred into B6 WT recipients by i.p. injection. The recipients of bm12 CD4+ T cells were injected with anti-SLAMF3, anti-SLAMF1 or isotype IgG1 (200mg/mouse) on days -1, 7, 14 and 21 days. Mice were euthanized on day 28. Anti-Chromatin, anti-ssDNA and anti-dsDNA in the serum of recipient mice were determined by ELISA is shown.
2.5
p=0.004
2
ns
1.5
Anti-Chromatin
1
0.5
0
aSLAMF3
isotype
aSLAMF1
5
p=0.002
4
ns
3
Anti-ssDNA
2
1
0
aSLAMF3
isotype
aSLAMF1
2.5
p=0.02
2
1.5
Anti-dsDNA
1
0.5
0
aSLAMF3
isotype
aSLAMF1
